# Outgrowth of a CSF3R-mutant clone drives a second myeloproliferative neoplasm in a chronic myeloid leukemia patient: a case report

**DOI:** 10.1186/s40364-021-00261-4

**Published:** 2021-01-30

**Authors:** Sarah A. Carratt, Diana Brewer, Julia E. Maxson, Brian J. Druker, Theodore P. Braun

**Affiliations:** 1 Knight Cancer Institute, Oregon Health & Science University, Portland, OR 97239 USA; 2 Division of Hematology and Medical Oncology, Oregon Health & Science University, Portland, OR 97239 USA

**Keywords:** Case report, Chronic myeloid leukemia, Chronic neutrophilic leukemia, Myeloproliferative neoplasm, Tyrosine kinase inhibitors, Clonal evolution

## Abstract

**Background:**

Chronic myeloid leukemia (CML) and chronic neutrophilic leukemia (CNL) are two myeloproliferative neoplasms with mutually exclusive diagnostic criteria. A hallmark of CML is the Philadelphia chromosome (Ph), which results in a BCR-ABL1 fusion gene and constitutive tyrosine kinase activity. CNL is a Ph-negative neoplasm and is defined in part by the presence of CSF3R mutations, which drive constative JAK/STAT signaling.

**Case presentation:**

Here, we report the exceedingly rare co-occurrence of two granulocytic myeloproliferative neoplasms in a 69-year old male patient. After an initial diagnosis of chronic myeloid leukemia, the patient’s clinical course was shaped by hematologic toxicity, the emergence of treatment-resistant BCR-ABL1 clones, and the expansion of a CSF3R-mutant clone without ABL1 mutations under selective pressure from tyrosine kinase inhibitors. The emergence of the CSF3R-mutant, neutrophilic clone led to the diagnosis of CNL as a second myeloproliferative neoplasm in the same patient.

**Conclusions:**

This is the first reported case of CNL arising subsequent to CML, which occurred under selective pressure from targeted therapy in a patient with complex clonal architecture. Patients with such molecularly complex disease may ultimately benefit from combination therapy that targets multiple oncogenic pathways.

## Background

Myeloproliferative neoplasms (MPNs) are clonal hematologic malignancies in which a hematopoietic stem cell defect drives proliferation of mature myeloid cell lineages. Chronic myeloid leukemia (CML) and chronic neutrophilic leukemia (CNL) are two MPNs with mutually exclusive diagnostic criteria. A hallmark of CML is the presence of the Philadelphia (Ph) chromosome, which results from t (9;22) and separates CML from Ph-negative MPNs: essential thrombocythemia, primary myelofibrosis, polycythemia vera, chronic neutrophilic leukemia [1]. The BCR-ABL1 fusion gene results from t (9;22) and drives the proliferation of mature myeloid cells through constitutive tyrosine kinase activity [2, 3]. In this report, we describe a second rare MPN, chronic neutrophilic leukemia (CNL), arising after a CML patient achieved a complete molecular remission. This is the first reported case of CNL arising subsequent to CML.

## Case presentation

A 69-year-old presented initially to his primary care provider with a progressive cough accompanied by weakness, fatigue, and abdominal distension. His white cell count was 113,900/mm^3^, hemoglobin was 11.8 g/dL, and platelet count was 325,000/mm^3^. A bone marrow biopsy showed a markedly hypercellular marrow (100%) with ~ 4% blasts, consistent with chronic phase CML. A 300 cell differential showed left-shifted myelopoiesis in which mid-range differentiated granulocytes—myelocytes, metamyelocytes with lesser amounts of promyelocytes—predominated over more differentiated forms. Myeloid and erythroid precursors were present in a 20:1 ratio. Auer rods were not observed. A Fluorescent In Situ Hybridization (FISH) assay confirmed a BCR-ABL1 translocation and cytogenetics revealed the Ph chromosome resulting from a (9;22) translocation. The major BCR-ABL1 transcript (p210) was 66.45% on the international scale (IS). The patient was started on 300 mg of nilotinib twice daily, which reduced his BCR-ABL1 to 0.403% (IS) in 18 weeks. However, the emergence of six new pathogenic mutations as well as hematologic toxicity of ABL1 inhibitors in this case made the treatment course complex (Tables 1, 2).

**Table 1:** Treatment strategy. The patient had difficulty tolerating TKIs due to hematologic toxicity (thrombocytopenia). Additionally, identification of three genetic variants triggered three treatment changes: imatinib and nilotinib-resistant BCR-ABL1 E255V, ponatinib-sensitive BCR-ABL1 T315I, ruxolitinib-sensitive CSF3R mutations. Dates initiated and discontinued are the number of weeks post-diagnosis of CML

Inhibitor	Molecular Target	Initiated	Discontinued	Reason Discontinued
Nilotinib	BCR-ABL1	0w	28w	Thrombocytopenia
Imatinib	BCR-ABL1	33w	78w	BCR-ABL1 p.E255V
Dasatinib	BCR-ABL1	79w	118w	BCR-ABL1 p.T315I
Ponatinib	BCR-ABL1	128w		
Ruxolitinib	CSF3R	252w

**Table 2:** Variant allele frequencies and BCR-ABL transcript levels 3 years apart for predicted pathogenic mutations. Outgrowth of a CSF3R-mutant clone during treatment with BCR-ABL1 tyrosine kinase inhibitors drives disease evolution from CML to CNL

Gene	Variant	COSMIC v92 FATHMM prediction	Variant Allele Frequency		
			69 weeks	116 weeks	225 weeks
KMT2C/MLL3	S1860C	Pathogenic (score 0.90)	50.62		48.92
CSF3R	T618I	Pathogenic (score 0.96)	11.58		41.09
CSF3R	W818*	Pathogenic (score 0.83)	10.22		40.26
TET2	R1167K	Pathogenic (score 0.99)	2.86		
ABL1	E255V	(not annotated)	2.01	Not detected (targeted PCR)	
ABL1	T3151	(not annotated)		Detected (targeted PCR)	
BCR-ABL1			2.50% (IS)	0.508% (IS)	0.000% (IS)

After 18 weeks on nilotinib, the patient presented with chest pain and was found to have pericarditis, atrial fibrillation, and severe thrombocytopenia (platelets at 11,000). The patient was ultimately started on imatinib at 400 mg/day but thrombocytopenia remained problematic. After 2 months, imatinib was reduced to 300 mg/day in an attempt to stabilize the patient’s ongoing thrombocytopenia. His platelets only slightly improved, however BCR-ABL1 transcripts decreased. The 400 mg/day dose was resumed when BCR-ABL1 rose from 0.68 to 2.74% (IS); however, subsequent ABL sequencing detected an imatinib and nilotinib-resistant E255V mutation [4], and the patient switched to dasatinib at 100 mg/day.

The leukemia responded well to dasatinib, though BCR-ABL1 transcript level plateaued above 0.1% (IS), the threshold for a major molecular response. ABL sequencing after 9 months of treatment detected a T315I mutation, which confers resistance to dasatinib, nilotinib, imatinib and bosutinib [5–11]. The patient was switched to ponatinib (30 mg/day) and his BCR-ABL1 transcript levels decreased steadily, falling below 0.1% (IS) after 3 months. The patient tolerated ponatinib well, with no thrombocytopenia. After 2 years on ponatinib, the (9;22) translocation was not detected by FISH in 200 cells scored for BCR (22Q11.2)/ABL (9Q34). Additionally, the BCR-ABL1 fusion gene was no longer detectable by PCR, 0.000% (IS). After 2.5 years, the dose of ponatinib was reduced to 15 mg/day.

**Fig. 1:**
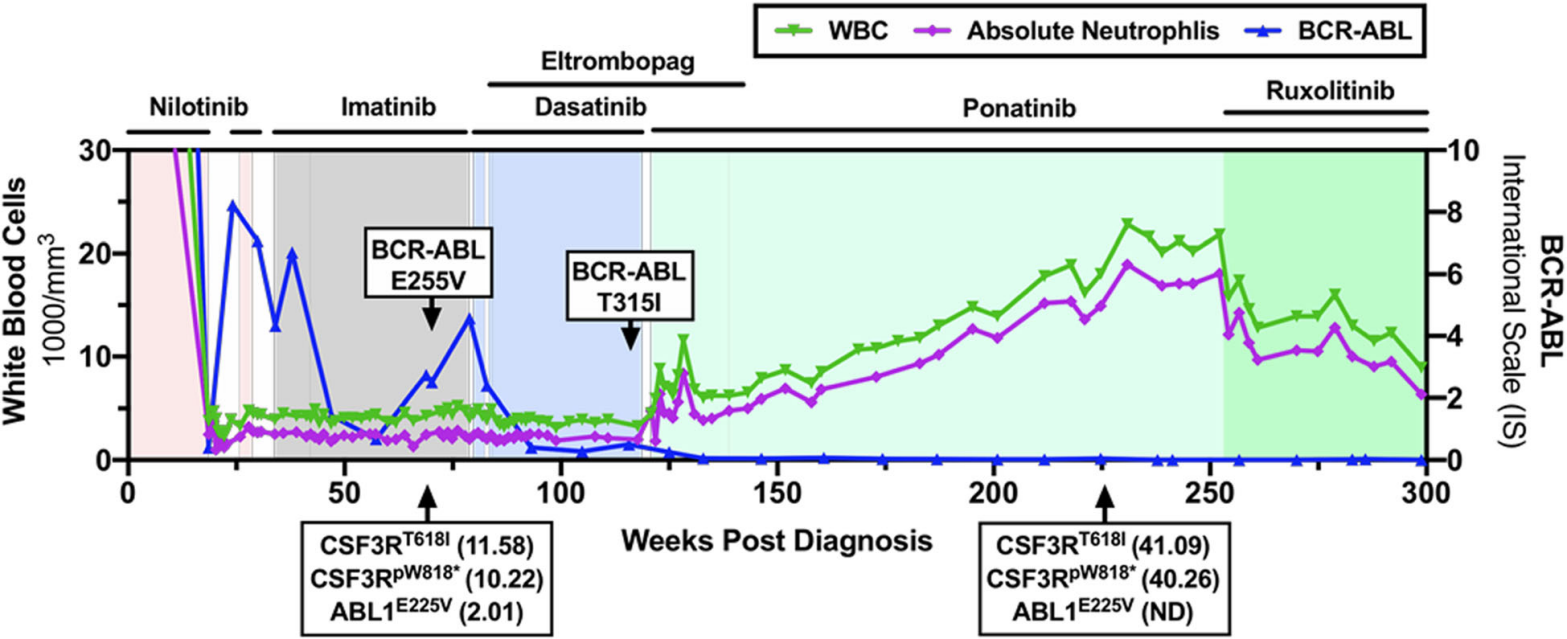
Emergence of a CSF3R-mutant, neutrophilic leukemia during targeted therapy for BCR-ABL1. Hematologic toxicity and the emergence of treatment-resistant clones shaped this CML patient’s clinical course. Ultimately, treatment with ponatinib successfully controlled the BCR-ABL1 clone, while another neutrophilic clone containing CSF3R mutations, but not BCR-ABL1, expanded

Despite the disappearance of the Ph chromosome, the patient’s white blood cell and absolute neutrophil counts rose while on ponatinib, raising concerns about whether a second Ph-negative MPN could be emerging (Fig. 1). A 220 gene next generation sequencing panel was ordered and two CSF3R mutations, T618I and W818*, were identified at a variant allele frequency (VAF) of 41.1 and 40.3%, respectively. Predicted pathogenic variants were identified based on the COSMIC v92 (Catalogue of Somatic Mutations in Cancer) database. Retrospective analysis of an archived marrow sample revealed that the CSF3R mutations had been present for at least the past 3 years. Bone marrow from diagnosis was not available for retrospective next generation sequencing analyses. In total, six predicted-pathogenic variants were detected in the patient over the course of his treatment, including two CSF3R mutations (T618I and W818*), two ABL1 mutations (E255V and T315I), KMT2C/MLL3 (S1860C), and TET2 (R1167K) (Table 2).

CSF3R mutations are a hallmark of chronic neutrophilic leukemia (CNL), a rare MPN defined by persistent mature neutrophilic leukocytosis. When the CSF3R variants were detected, the patient’s WBC was 22,800/mm^3^ with 83% neutrophils, within the range of WHO-defined CNL (11,000–126,000/mm^3^, median 39,000/mm^3^). The patient was started on ruxolitinib (5 mg twice daily), which demonstrated efficacy against CNL and CSF3R-mutant atypical CML in a recent clinical trial [12, 13]. With ruxolitinib, the patient’s WBC count decreased slightly and stabilized between 12,800-17,400/mm^3^, absolute neutrophils between 9700 and 14,300/mm^3^, platelets were stable at 124,000-155,000/mm^3^, hemoglobin at 15.3–16.6 g/dL, hematocrit at 46.6–51%. Ruxolitinib was increased to 7.5 mg twice daily after 7 months, which lowered his WBC (8900-13,000/mm^3^) and absolute neutrophil (6300-10,000/mm^3^) counts further. At 5.8 years post CML diagnosis and 3.5 years on ponatinib, a third FISH analysis was performed and the (9;22) translocation was still not detectable (200 nuclei).

## Discussion and conclusions

This patient had difficulty tolerating tyrosine kinase inhibitors (TKIs), primarily due to hematologic toxicity. While thrombocytopenia is a known side effect of TKIs, the severity and protracted nature of the side effect in this instance suggests dysfunction in the residual BCR-ABL1 negative stem cells. In this case, the emergence of six predicted-pathogenetic variants over the five-year course of treatment suggests that the patient either had a markedly elevated mutational load prior to diagnosis—with different clones emerging in response to TKI selection—or a propensity to develop mutations beyond what is normally associated with aging.

The mutations in BCR-ABL1 and TET2 were present at similar variant allele frequencies at 69 weeks and lost during treatment with dasatinib and ponatinib, suggesting they were present in the same clone. Meanwhile, the CSF3R-mutant clone expanded during BCR-ABL1-directed therapy and is likely independent. Given that the increase in neutrophils began when the patient was switched from dasatinib to ponatinib, it is plausible that the expansion of this clone occurred during treatment with ponatinib specifically. It is known that CSF3R truncating mutations are sensitive to dasatinib [14], and that may have contributed to a delayed onset of this second MPN.

It is well established that selective pressure from TKIs allows for the outgrowth of BCR-ABL1 clones harboring point mutations in ABL1. However, this study suggests that the clearance of a BCR-ABL1 positive clone allowed for the outgrowth of a second, genetically distinct leukemia. It is interesting to note that two granulocytic leukemias arose in this patient, suggesting that either cell intrinsic or microenvironmental factors caused a specific predilection for malignancies of the granulocytic lineage. Germline mutations have not been accessed for this patient. This case describes the exceedingly rare co-occurrence of CML and CNL in the same individual and is an unusual example of clonal evolution under selective pressure from targeted therapy. These findings highlight that myeloid disease with complex clonal architecture is prone to molecular evolution. Patients with such molecularly complex disease may ultimately benefit from combination therapy that targets multiple oncogenic pathways.

## Data Availability

Data sharing requests should be sent to the corresponding author.

## Abbreviations

MPNs: Myeloproliferative neoplasms
CML: Chronic myeloid leukemia
CNL: Chronic neutrophilic leukemia
Ph: Philadelphia
FISH: Chromosome, Fluorescent In Situ Hybridization
IS: International scale
TKIs: Tyrosine kinase inhibitors
